# Adverse childhood experiences in patients with psoriasis

**DOI:** 10.47626/2237-6089-2021-0251

**Published:** 2022-07-14

**Authors:** Akina Andreia Akamine, Gabriela De Souza Rusch, Renato Nisihara, Thelma L. Skare

**Affiliations:** 1 Faculdade Evangélica Mackenzie de Medicina Curitiba PR Brazil Faculdade Evangélica Mackenzie de Medicina, Curitiba, PR, Brazil.

**Keywords:** Psoriasis, mental health, autoimmunity

## Abstract

**Introduction:**

Adverse childhood experiences (ACEs) have been linked to occurrence of autoimmune diseases in adults, including psoriasis.

**Objectives:**

To study the prevalence of ACEs in psoriasis patients, comparing them with a sample from the general population.

**Methods:**

Three hundred and eighteen individuals were included (104 psoriasis patients and 214 controls). Patients and controls answered questions on an ACE study questionnaire about experiences of childhood abuse, negligence, domestic violence, and household dysfunction. Questionnaire scores range from zero (best result) to 8 (worst scenario). Psoriasis patients’ charts were reviewed for epidemiological, clinical, and treatment data. A Psoriasis Area Severity Index (PASI) was calculated from measurements taken when the questionnaire was administered.

**Results:**

Psoriasis patients reported a median of 4 ACEs (interquartile range [IQR] = 3-5) while controls had a median of 3 (IQR = 2-4) with p < 0.0001. The number of ACEs was not associated with PASI, age of disease onset, or presence of associated arthritis (all p > 0.5). Female psoriasis patients had more ACEs than males (p = 0.04).

**Conclusion:**

Patients with psoriasis have more ACEs than controls and ACEs were more common in female patients.

## Introduction

Psoriasis is a chronic skin disorder that affects 1.5-2% of the general population in industrialized countries^1^ and expresses itself clinically as well-defined, red plaques covered by silver scales.^2^ Emotional, environmental, and autoimmune events have been associated with its occurrence.^2-4^

Psoriasis patients may suffer from several psychological problems such as anxiety, depression, alexithymia, poor self-esteem, and suicidal ideation.^1,3^ Currently, psoriasis is considered to be a psycho-dermatological illness, i.e. a physical disease that can be caused or intensified by psychological events.^3^ Supporting this hypothesis, psychosocial factors have been identified in 40-80% of psoriasis onset or flares.^5^

Experiencing traumatic events and psychological suffering that exceed the individual’s capacity to cope may be interrelated to onset, worsening, and/or reoccurrence of several autoimmune disorders.^3^ It is hypothesized that the increasing prevalence of autoimmune diseases during recent decades in industrialized countries should be at least partially attributed to high stress levels.^6^ During stress response, rising levels of catecholamines and glucocorticoids affect the function of several immune cells and modify the profile of cytokines released.^7^

Childhood traumatic experiences have been connected to the appearance of several autoimmune disorders^3^ and to increased inflammatory markers such as C reactive protein levels into adulthood.^8^ Simonic´ et al.,^3^ studied 100 psoriasis patients, finding that they had higher prevalence of negative traumatic experiences during developmental periods than controls.

The influence of these events can be affected by social, cultural, and individual patient characteristics. Some studies reported high prevalence of adverse childhood events (ACEs) in regions with low income.^9,10^

Herein we studied ACEs in a sample of Brazilian patients with psoriasis to determine their frequency and associations with clinical variables.

## Methods

All procedures performed in this study were in accordance with the ethical standards of the institutional research committee and with the 1964 Helsinki declaration and its later amendments or comparable ethical standards. This cross-sectional study was approved by the Research Ethics Committee at Faculdade Evangélica Mackenzie de Medicina, Curitiba, PR, Brazil. All participants signed written informed consent.

The study recruited 318 individuals (104 psoriasis patients and 214 controls). This was a convenience sample that comprised all psoriasis patients who came for regular consultations to a single Dermatology Unit over the period of one year and who agreed to participate in the study. Controls were self-declared healthy individuals recruited from among patients’ companions and individuals who came to the Ophthalmology Clinic for refraction testing. Individuals less than18 years old or with cognitive impairment were excluded as were those with any other autoimmune disorder. All patients enrolled were Brazilians and the language used was Brazilian Portuguese.

Patients and controls answered a questionnaire on epidemiological data (that covered age, sex, income, years of formal study, and religion) and answered the ACEs Study Questionnaire. This instrument encompasses questions on childhood traumatic events and covers eight domains: emotional abuse, physical abuse, violent treatment by mother, use of substances/alcohol at home, sexual abuse, mental disease at home, jailed family member and parental divorce.^11^ The questionnaire score ranges from zero (best result) to 8 (worst scenario). The questionnaire used comprises questions selected by Soares et al.^12^ from those on an ACE questionnaire^11^ translated and validated in Brazilian Portuguese by Grassi-Oliveira et al.^13^

Psoriasis patients had their charts reviewed for disease duration, psoriasis subset, involvement of nails and scalp, and presence of arthritis. The extent of skin disease was evaluated using the Psoriasis Area Severity Index (PASI),^14^ measured at the same time as the questionnaire was administered.

Frequency and contingency tables were constructed. Data distribution was studied with the Shapiro Wilks test. Central tendency was expressed as median and interquartile range (IQR). Fisher’s and chi-squared tests were used to compare nominal data and the unpaired *t* test and Mann Whitney test were used for numerical data comparisons. The Spearman test was used to analyze correlation between PASI and number of ACEs. The significance level adopted was 5%.

## Results

### Description of the study sample

In the group of 104 psoriasis patients; 26 (25%) were men and 78 (75%) were women, median age was 43 years (IQR = 35-43), and median age at disease onset was 23 years (IQR = 16-35). The scalp was involved in 75/104 (72.1%); nails in 49/104 (47.1%), and arthritis was present in 42/104 (40.3%). Psoriasis subsets present in this sample were: plaque psoriasis in 72/98 (73.4%), palmoplantar in 12/98 (12.2%), guttate in 11/98 (11.2%), inverse in 1/98 (1.0%), and erythrodermic in 2/98 (2.0%). In this sample, 3/104 (2.8%) individuals used cyclosporin, 7/104 (6.7%) used acitretin; 39/104 (37.5%) used methotrexate, 19/104 (18.2%) used anti-TNF alpha, 3/104 (2.8%) used secukinumab, and 1/104 (0.9%) used ustekinumab. Topical medication was the only treatment used by 27/104 (25.9%). In this sample, PASI scores ranged from 0 to 32, with a median value of 2.4 (IQR = 1.5 to 5.9).

### Comparison of psoriasis patients with controls

Table 1 lists the results of the comparison of psoriasis patients with controls.

**Table 1:** Pairing data and comparison of ACEs in psoriasis patients with controls

	Psoriasis patients n = 104	Controls n = 214	p
Age (years)*	43 (35-53)	53.5 (22-62)	0.83
Sex			0.51
Females	78 (75)	153 (71.4)	
Males	26 (25)	61 (28.5)	
Religion			0.45
None	14	42	
Catholic	59	53	
Evangelic	24	109	
Others	7	10	
Income (multiples of minimal wage)			
Up to 3x/month	51/79 (64.5)	115/163 (70.5)	0.34
More than 3x/month	28/79 (35.45)	48/163 (29.4)	
Years of formal study*	11 (7.2-15)	14 (4-16)	0.07
ACEs*	4 (3-5)	3 (2-4)	< 0.0001
ACE subtypes (categorical)			0.001
None	3 (2.8)	10 (4.6)	
One	6 (5.7)	40 (18.6)	
Two	14 (13.4)	42 (19.6)	
Three	18 (17.3)	47 (21.9)	
Four	24 (23.0)	34 (15.8)	
Five	20 (19.2)	23 (10.7)	
Six	14 (13.4)	13 (6.0)	
Seven	5 (4.8)	4 (1.8)	
Eight	0	1 (0.4)	

Data presented as n or n (%), unless otherwise specified.

ACEs = adverse childhood events.

* Median (interquartile range).

Table 2 shows the results for each form of ACE analyzed individually. About 81/104 (77.8%) of psoriasis patients and 109/214 (50.9%) of the control sample reported 3 or more ACEs.

**Table 2:** Comparison of individual adverse childhood events in psoriasis patients and controls

	Psoriasis n = 104	Controls n = 214	p
Emotional abuse	85 (81.7)	165 (77.1)	0.38
Physical abuse	64 (61.5)	86 (40.1)	0.0004 (OR = 2.3;95%CI = 1.4-3.8)
Violent treatment by the mother	58 (55.7)	84 (39.2)	0.006 (OR = 1.9;95CI = 1.2-3.1)
Substance/alcohol use at home	80 (76.9)	132 (61.6)	0.007 (OR = 2.04;95%CI = 1.2-3.4)
Mental disease in the family	41(39.4)	79 (36.9)	0.71
Sexual abuse	25 (24.0)	20 (9.3)	0.0005 (OR = 3.0;95%CI = 1.6-5.8)
Jailed family member	14 (13.4)	21 (9.8)	0.33
Parental divorce	36 (34.6)	38 (17.7)	0.0008 (OR = 2.4;95%CI = 1.4-4.2)

Data presented as n (%).

### Study of psoriasis variables with ACEs

A comparison of the number of ACEs in psoriasis patients with arthritis compared to the number in those without arthritis returned p = 0.56. A comparison of patients using topical medications versus those on systemic drugs for treatment was also non-significant (p = 0.61).

A comparison of the number ACEs in patients with disease onset at < 30 years of age with those whose onset was at ≥ 30 years of age returned p = 0.44. The female subset of the psoriasis sample had a median of 4 ACEs (IQR = 3-5) and the male subset had 3 (IQR = 2-4), with p = 0.04.

Analysis of the correlation between PASI and number of ACEs revealed p = 0.89; rho = -0.01; and 95%CI = -0.29 to +0.26.

## Discussion

The analysis of this sample of Brazilian psoriasis patients showed that occurrence of psoriasis is linked to a high number of ACEs. These results agree with previous findings that patients with psoriasis^3^ or with psoriatic arthritis^15^ reported higher numbers of traumatic experiences during childhood than the general population.

The mechanisms underlying this association are unknown. Harsh family environments may stimulate a proinflammatory phenotype that persists through adulthood; stress-driven sympathoadrenal activation increases expression of pro-inflammatory cytokines.^16^ Other possibilities include recall bias in psoriasis patients or ACEs affecting obesity, which in turn affects risk of psoriasis.^17^

The elevation of CRH seen in the context of chronic stress is interesting from the pathophysiologic view of psoriasis. CRH stimulates cutaneous production of cytokines like IL-6 and IL-11 and amplifies expression of adhesion molecules and production of transcription factor NF-𝜅B (nuclear factor kappa-light-chain-enhancer of activated B cells), shifting keratinocytes into an immune active state.^16^ CRH receptors are increased in psoriasis compared to normal skin, and expression of CRH-R1 (CRH receptor type 1) is correlated with PASI.^18^

We could not prove any links between number of ACEs and age of disease onset, severity of skin disease measured by PASI, or presence of associated arthritis. Moreover, patients requiring systemic treatment, which can be considered as a marker for more severe disease, had similar number of ACEs to those not on systemic treatment. Other investigators have studied the association between extent of skin disease and psychological disturbance and also failed to prove any association.^3,15^

Psoriasis is as frequent in males as in females in several countries, including in Brazil,^19^ but the female subset of our psoriasis sample had higher numbers of ACEs than the male subset. In Brazil, adolescent females have more adverse experiences than males and the most common ACE was parental separation, followed by emotional neglect, and domestic violence.^20^ The most common current ACEs in both psoriasis and control samples were emotional abuse and use of substance/alcohol at home.

Children who have suffered ACEs have a tendency to become dysfunctional adults and this may influence their parenting practices; they may reproduce the problem in the next generation.^21^ It is therefore important to identify them in order to intervene in this vicious cycle. The knowledge that psoriasis patients may be at risk of having higher numbers of ACEs offers the attending physician an opportunity to identify this situation and to refer these patients to receive adequate support to interrupt perpetuation of the cycle.

The present study has several limitations. The low number of participants and their low PASI are some of these. Another is the study design that relies on the patients’ memory to recall ACEs. However, the study has the merit of revealing the possible association between psoriasis and ACEs.
